# Synergistic protective effects of rhizobacterial culture filtrate and zinc oxide nanoparticles against *Pantoea* leaf spot in cucumber

**DOI:** 10.1186/s12870-025-07925-5

**Published:** 2025-12-29

**Authors:** Amira A. Helaly, Amany H. M. Shams, Ibrahim A. A. Mohamed, Eman F. A. Awad-Allah

**Affiliations:** 1 Vegetable Crops Department, Faculty of Agriculture, Alexandria University, Alexandria, 21545 Egypt; 2 Plant Pathology Department, Faculty of Agriculture, Alexandria University, Alexandria, 21545 Egypt; 3 Botany Department, Faculty of Agriculture, Fayoum University, Fayoum, 63514 Egypt; 4 Soil and Water Sciences Department, Faculty of Agriculture, Alexandria University, Alexandria, 21545 Egypt

**Keywords:** Antimicrobial activity, Bacterial leaf spot (BLS), Biostimulants, Biotic stress, Cucumber, Nanofertilizer, Secondary metabolites, Sustainability

## Abstract

Global food security is increasingly threatened by plant diseases and the excessive use of chemical pesticides and fertilizers, highlighting the urgent need for sustainable and environmentally friendly alternatives. Nanotechnology and biological agents such as plant-growth-promoting rhizobacteria (PGPR), particularly *Bacillus* spp., offer cutting-edge strategies for sustainable crop protection. This study investigates the individual and synergistic effects of *Bacillus* culture filtrate (BCF) and zinc oxide nanoparticles (ZnO-NPs) in controlling *Pantoea* sp., the causal agent of bacterial leaf spot (BLS) in cucumber, and enhancing plant performance under greenhouse conditions. Antimicrobial activity was assessed in vitro, *Bacillus*-derived secondary metabolites were identified via gas chromatography–mass spectrometry (GC–MS), and plant health indicators—including disease severity, physiological traits, and fruit yield—were evaluated under greenhouse conditions. The in vitro assays revealed that ZnO-NPs exhibited a concentration-dependent antibacterial effect, with inhibition zones increasing from 3 mm at 0 ppm to 38 mm at 200 ppm. Meanwhile, *Bacillus* culture filtrate alone produced a significant inhibition zone of 40 mm against *Pantoea* sp. GC–MS analysis of the methanolic extract of *Bacillus* sp. identified twenty bioactive compounds, many of which are known for their antimicrobial and plant growth-promoting properties, likely contributing to the observed effects. Under greenhouse conditions challenged with *Pantoea* sp., the co-application of 200 ppm ZnO-NPs and *Bacillus* culture filtrate significantly reduced bacterial leaf spot severity in cucumber plants, lowering the symptom score to 1.1 on a 1–7 scale. This combined treatment also significantly enhanced physiological traits, including leaf chlorophyll content (SPAD value: 59.0), total phenolic content (1.76 mg GAE g^−1^ FW), and leaf macro- and micronutrient levels, while maximizing productivity—achieving the highest fruit number per plant (14.2) and total yield (2.42 kg/plant). These findings support the integration of ZnO-NPs and *Bacillus*-based biostimulants as a promising eco-friendly strategy for disease management and productivity enhancement in cucumber cultivation.

**Graphical abstract:**

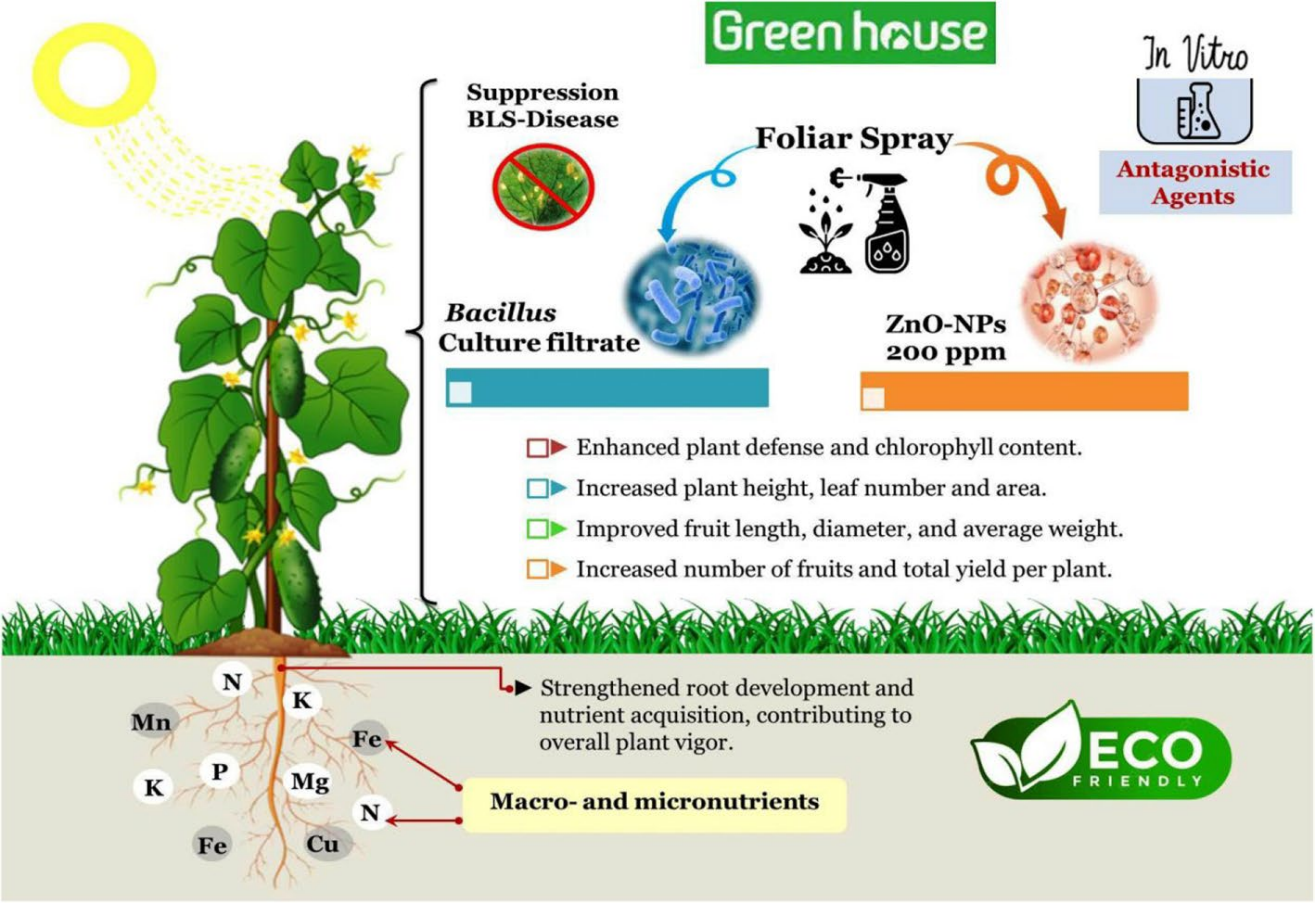

## Introduction

*Pantoea* species are Gram-negative, yellow-pigmented, rod-shaped bacteria belonging to the class *Gammaproteobacteria* and the family *Enterobacteriaceae*. They exhibit remarkable ecological plasticity, enabling them to inhabit diverse environments including soil, water, insects, and plant tissues [1, 2]. Within this genus, strains may occur as non-pathogenic endophytes, whereas others are recognized as phytopathogens capable of causing economically significant diseases in a wide range of crops such as maize, rice, wheat, strawberries, onions, eucalyptus, and melons [1, 3, 4]. Disease symptoms caused by *Pantoea* spp. vary considerably and may include leaf blight, leaf spot, grain discoloration, stem necrosis, and bulb decay [2]. Numerous reports have confirmed the phytopathogenic potential of *Pantoea* spp., including white spot in maize in Brazil [5], leaf blight in rice in India [6], strawberry blight in Italy and Egypt [3, 7], and leaf spot in maize in Mexico and Poland [8, 9]. Recently, *Pantoea* species have also been reported to cause leaf spot in cucumber in Hainan, China, where infected plants exhibited yellow–brown, water-soaked lesions that expanded to cause complete leaf necrosis [10]. The increasing frequency of *Pantoea*-associated outbreaks highlights the growing importance of this genus as a global plant pathogen. Favorable infection conditions include high humidity and temperature, particularly under greenhouse environments, where bacteria may enter through natural plant openings or wounds and can be disseminated via contaminated seeds or rain splash [11]. Despite global advances in understanding the pathogenicity of *Pantoea* spp., their occurrence in cucumber cultivation remains insufficiently documented, particularly in North Africa. In this context, the present study reports, to our knowledge, the first observation of *Pantoea* sp.-associated bacterial leaf spot in cucumber (*Cucumis sativus* L.) under greenhouse conditions in Egypt, based on naturally infected seedlings from a private farm in Borg El-Arab, Alexandria Governorate. Given the increasing incidence of *Pantoea*-induced diseases, especially under greenhouse conditions, and ongoing concerns regarding resistance development and environmental impacts of chemical pesticides, there is an urgent need for sustainable and proactive management strategies, including biological agents and nanomaterials [11].

Nanotechnology has emerged as a promising tool for sustainable agriculture, offering eco-friendly alternatives to conventional agrochemicals while enhancing crop productivity and resilience [12]. Due to their small size, high surface area, solubility, and surface reactivity, nanomaterials—particularly zinc oxide nanoparticles (ZnO-NPs)—facilitate efficient nutrient uptake and targeted delivery within plant systems [12]. Zinc (Zn), an essential micronutrient, plays a crucial role in plant immunity as a component of metalloenzymes involved in hormonal regulation and microbial defense responses [13]. Accordingly, the use of ZnO-NPs as nano-nutrition is gaining attention for its dual capacity to mitigate biotic and abiotic stresses, offering innovative solutions for crop health and food security [13]. Parallel to advances in nanotechnology, plant growth-promoting rhizobacteria (PGPR) naturally colonize plant roots and contribute to host fitness under stress conditions [14–16]. PGPR promote plant health through enhanced nutrient acquisition, synthesis of stress-alleviating biomolecules, and direct antagonism against pathogens via antibiotic production and nutrient competition [14]. Species within the genus *Bacillus* are particularly valued for their robust biocontrol and growth-promotion capacities, largely due to their ability to produce diverse extracellular secondary metabolites [14]. Moreover, the application of *Bacillus* culture filtrate (BCF), which contains extracellular bioactive metabolites, represents an eco-friendly alternative to live-cell inoculation, overcoming limitations related to field instability and microbial interactions commonly encountered with live *Bacillus* treatments [14].

Although numerous studies have examined the individual roles of *Bacillus* spp. and zinc oxide nanoparticles (ZnO-NPs) in promoting plant growth and enhancing tolerance to abiotic stress, their combined potential in managing bacterial leaf spot disease caused by *Pantoea* sp. in cucumber remains largely unexplored. Therefore, this study aims to evaluate the individual and synergistic effects of *Bacillus* culture filtrate and ZnO-NPs on disease suppression, plant growth, physiological responses, and fruit yield under greenhouse conditions. Additionally, we characterize the antimicrobial activity of these treatments in vitro and profile *Bacillus*-derived secondary metabolites using gas chromatography–mass spectrometry (GC–MS).

## Materials and methods

The current study was conducted both in vitro and under greenhouse conditions at the Plant Pathology Department, Faculty of Agriculture, Alexandria University, El-Shatby, Alexandria, Egypt (approx. GPS: 31.206134 N, 29.919707 E).

### Collection and isolation of the pathogen and antagonistic isolates

#### Isolation of the causal agent of the disease

Cucumber leaves exhibiting characteristic bacterial leaf spot symptoms—small, water-soaked, yellow–brown lesions—were collected from symptomatic seedlings grown in a greenhouse on a private farm in Borg El-Arab, Alexandria Governorate, Egypt, with prior permission from the farm owner. Approximately 1 cm^2^ leaf sections were excised from the interface of necrotic and healthy tissues, surface-sterilized in 70% ethanol for 3 min, rinsed with sterile distilled water for 1 min, and air-dried under aseptic conditions.

**Fig. 1:**
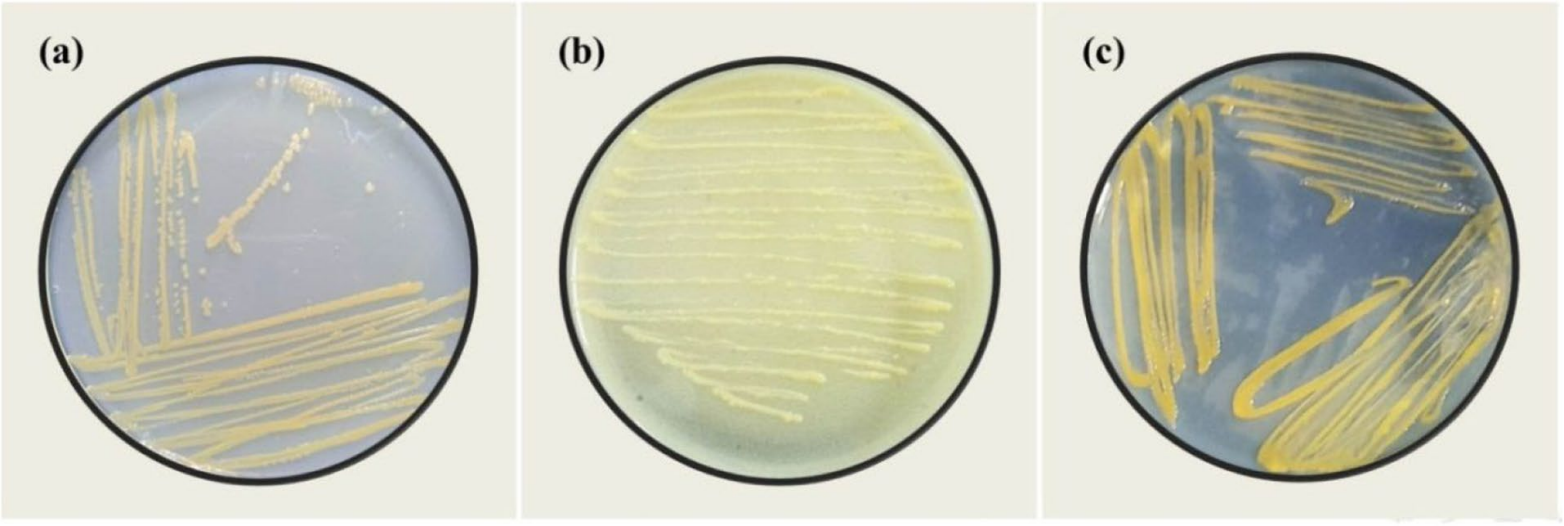
Colonial morphology of the isolated pathogen on: (**a**) Nutrient Agar (NA), (**b**) Yeast Extract–Dextrose–Calcium Carbonate Agar (YDC), and (**c**) PA 20 semi-selective medium

The sterilized tissues were macerated and serially diluted in 0.8% NaCl solution. Appropriate dilutions were plated onto Nutrient Agar (NA) and incubated at 29 °C for 24–48 h [10]. To improve selectivity and facilitate purification of the bacterial pathogen, representative colonies were sub-cultured onto Yeast Extract–Dextrose–Calcium Carbonate Agar (YDC) [17], and PA 20 semi-selective medium [18, 19]. Colonies exhibiting distinct morphological characteristics on the three media were isolated and purified for further identification (Fig. 1).

#### Isolation of the antagonistic isolate

**Fig. 2:**
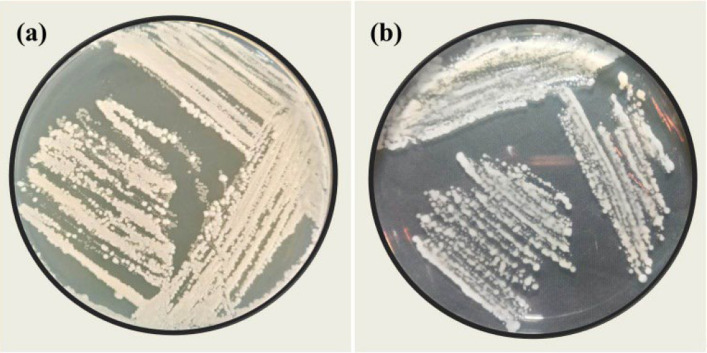
Rhizobacterial colony isolated on Nutrient Agar (NA) from cucumber rhizosphere: (**a**) Initial colony development from soil dilution plating; (**b**) purified isolates after sub-culturing

*Bacillus* sp. was isolated from the rhizosphere of healthy, vigorous cucumber plants grown in various open fields in Alexandria Governorate, Egypt. Five soil samples were collected from a depth of 5–15 cm after removing the top 3 cm of surface soil. From each sample, 1 g of soil was used for serial dilution. Aliquots (100 µL) from dilutions of 1 × 10^–6^, 1 × 10^–7^, and 1 × 10^–8^ CFU mL^−1^ were plated onto Nutrient Agar (NA) and incubated at 28 ± 2 °C for 24 h, following the method of Islam et al. [20]. Bacterial colonies exhibiting distinct morphological characteristics were selected and purified for further evaluation (Fig. 2).

### Morphological and biochemical characterization of bacterial isolates

Morphological and biochemical characterization was conducted for both the pathogenic and antagonistic isolates using purified cultures, following standard procedures outlined in *Bergey's Manual of Determinative Bacteriology* [21].

For the pathogenic isolate, the following tests were performed: Gram reaction using the 3% KOH solubility method, catalase activity, indole production via Kovac’s reagent [22], oxidase test (to detect cytochrome oxidase), nitrate reduction, arginine dihydrolase activity, gelatin hydrolysis [23], hydrogen sulfide (H_2_S) production, and the hypersensitive reaction (HR) on tobacco leaves [24].

For the antagonistic isolate, colony morphology (color, shape, texture) was recorded. The Gram reaction was determined via the KOH solubility test. Catalase and oxidase activities were assessed according to the method described by Rajat et al. [25] to evaluate their metabolic adaptation to aerobic or anaerobic conditions.

According to Table 1, the pathogenic bacterial isolate produced yellow, smooth, round colonies on Nutrient Agar and was Gram-negative. It tested positive for catalase, oxidase, indole production, nitrate reduction, arginine dihydrolase, gelatin hydrolysis, H_2_S production, and elicited a clear hypersensitive reaction on tobacco leaves, confirming its pathogenicity.

**Table 1:** Morphological and biochemical characteristics of the pathogenic and antagonistic isolates

Test	Pathogenic Isolate	Antagonistic Isolate
Colony morphology	Yellow, smooth, round	Creamy white, mucoid, circular
Gram reaction (KOH test)	Negative	Positive
Catalase	+	+
Oxidase	+	+
Indole production	+	ND
Nitrate reduction	+	ND
Arginine dihydrolase	+	ND
Gelatin hydrolysis	+	ND
H₂S production	+	ND
Hypersensitive reaction on tobacco	+ (HR⁺)	–
Pathogenicity test on cucumber leaves	+	–

“ + ” = positive; “–” = negative; “*ND*” = not determined

To verify Koch’s postulates, a pathogenicity assay was conducted as follows: *Pantoea* sp. was cultured in nutrient broth at 28 °C, and cells in the logarithmic growth phase were harvested by centrifugation at 5,000 rpm for 2 min at 4 °C. The resulting pellet was resuspended in sterile distilled water to obtain a bacterial suspension with an OD600 of 0.5. The bacterial suspension was gently infiltrated into the abaxial surface of healthy cucumber leaves 18 days after transplanting (DAT) in the greenhouse using a sterile syringe, applying 100 μL (0.1 mL) per leaf. The syringe tip was carefully positioned between the veins on the lower leaf surface to ensure uniform infiltration. Sterile distilled water served as a negative control, following the procedure of Lao et al. [10]. To maintain optimal humidity and promote infection, inoculated plants were covered with transparent polyethylene bags for 48 h and kept under greenhouse conditions (21–27 °C) and observed daily for symptom development.

**Fig. 3:**
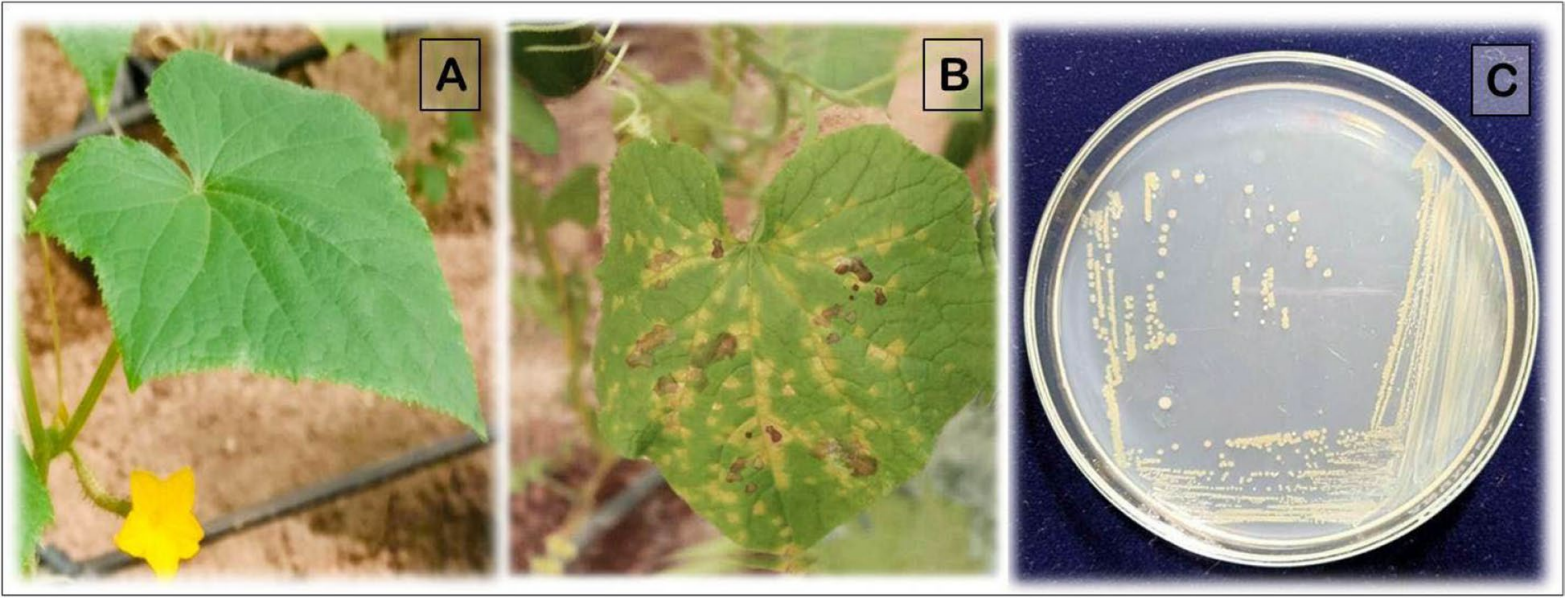
Pathogenicity test of *Pantoea* sp. on cucumber leaves under greenhouse conditions. **A** Healthy cucumber leaf (control) showing no symptoms. **B** Leaf inoculated with *Pantoea* sp. displaying characteristic water-soaked and necrotic lesions at 23 days post-inoculation (dpi). **C** *Pantoea* sp. colonies re-isolated from symptomatic cucumber leaves on nutrient agar (NA) medium, exhibiting typical yellow, smooth, round morphology

Early symptoms, characterized by slight water-soaking at the infiltration sites, appeared within 2–4 days post-inoculation (dpi). By 5–7 dpi, yellow necrotic spots developed and gradually expanded along the leaf veins. More pronounced necrosis and chlorotic regions became evident between 10 and 14 dpi, progressing to extensive gray necrosis and leaf yellowing by 14–23 dpi. No symptoms were observed on control leaves injected with sterile distilled water (Fig. 3A–B).

The pathogen was consistently re-isolated from symptomatic cucumber leaves, yielding colonies on nutrient agar (NA) medium with characteristics typical of *Pantoea* sp. (Fig. 3C), whereas no colonies were obtained from control leaves. The pathogen was further confirmed by molecular analysis, thereby fulfilling Koch’s postulates.

In contrast, the antagonistic isolate formed creamy white, circular, mucoid colonies and was Gram-positive. It showed positive catalase and oxidase activity, suggesting its adaptability to aerobic conditions (Table 1). Other pathogenicity-related biochemical tests were not determined for the antagonistic isolate.

### Molecular identification of the pathogen and antagonistic isolates

#### DNA extraction

Genomic DNA was extracted from overnight bacterial cultures grown in LB broth (1.5 mL) using a mini-preparation method as described by [26]. Cultures were centrifuged at 5,000 × g for 2 min, and the pellet was resuspended in 540 μL TE buffer (0.1 M Tris–HCl, 0.1 M EDTA, pH 8.0). Subsequently, 30 μL of 10% SDS was added, and the mixture was incubated at 37 °C for 60 min. Cell lysis was followed by the addition of 100 μL of 5 M NaCl and 80 μL of CTAB/NaCl solution, with further incubation at 65 °C for 10 min. DNA was extracted using 700 μL chloroform:isoamyl alcohol (24:1) at room temperature (approximately 25 °C), followed by centrifugation at 12,000 × g for 5 min, and the aqueous phase was transferred to a new tube. DNA was precipitated with 600 μL chilled isopropanol (− 20 °C for 30 min), centrifuged at 12,000 × g for 10 min, washed with 70% ethanol, air-dried, and resuspended in 20 μL TE buffer.

#### PCR amplification of 16S rRNA Gene

The 16S rRNA gene was amplified using universal primers 27 F (AGAGTTTGATCCTGGCTCAG) and 1492R (TACGGYTACCTTGTTACGACTT) as described by Dos Santos et al. [27]. PCR reactions (50 μL) contained 5 μL 10 × buffer, 2 μL of each primer (10 pmol), 4 μL MgCl_2_ (25 mM), 4 μL dNTPs (2.5 mM), 2 μL genomic DNA (50 ng), and 0.4 μL Taq DNA polymerase (5 U/μL; Promega, Germany). Amplification was performed in a thermal cycler (Techne, UK) with the following program: initial denaturation at 95 °C for 5 min; 34 cycles of 95 °C for 45 s, 50 °C for 1 min, and 72 °C for 2 min; followed by a final extension at 72 °C for 10 min.

#### 16S rRNA gene sequencing and identification

PCR products (1550–1600 bp) were purified using CentriSep spin columns and sent to Macrogen (South Korea) for sequencing. Sequence identities were confirmed via BLAST analysis on the NCBI database (https://www.ncbi.nlm.nih.gov). The 16S rRNA gene sequences were deposited in GenBank under accession numbers PX509262 for *Pantoea* sp. and PX509261 for *Bacillus* sp*.*

#### Alignment and phylogenetic analyses

Pairwise and multiple DNA sequence alignments were carried out in the GenBank database were achieved in BLASTN searches at the National Center for Biotechnology Information site (http://www.ncbi.nlm.nih.gov).

Alignments were made by using Molecular Evolutionary Genetics Analysis version 11.0.13 (MEGA11) software. Phylogenetic trees were constructed using neighbor joining method from the (CLUSTAL W) alignment [28]. The obtained sequences were compared with different international *Pantoea* sp. and *Bacillus* sp. obtained from GenBank.

### In vitro antagonistic activity of *Bacillus* culture filtrate against *Pantoea* sp

#### Preparation of *Bacillus *culture filtrate

*Bacillus* sp. was cultured in 250 mL Erlenmeyer flasks containing 150 mL of sterilized nutritional glucose (2%) broth medium (3 g beef extract, 5 g peptone, 20 g glucose/1L distilled water, pH 7.2). Flasks were inoculated with 24-h-old bacterial cultures and incubated at 28 ± 1 °C for 24–48 h. Following incubation, the cultures were centrifuged at 15,000 rpm for 20 min (E20B1, Centurion Scientific, UK). The bacterial pellet was diluted with sterile distilled water (SDW) and adjusted to make an OD600 of 0.6, corresponding to a final concentration of ca. 10^8^–10^9^ CFU ml^−1^. Before inoculation, the numerical density of bacterial cells was estimated [29]. The *Bacillus* culture filtrate (BCF) was prepared by centrifuging the culture at 10,000 rpm for 10 min, followed by collection of the supernatant and filtration through a 0.45 µm syringe filter.

#### Evaluation of antibacterial activity of *Bacillus* culture filtrate

The antagonistic activity of the *Bacillus* Culture Filtrate against *Pantoea* sp. was evaluated using the well diffusion method as described by Balouiri et al. [30]. Nutrient agar plates were inoculated using a swab spread method with *Pantoea* sp., and 4 mm wells were carefully created in the agar using a sterile cork borer. Well was filled with 100 µL of the *Bacillus* culture filtrate, while SDW served as a negative control. Plates were incubated at 28 °C for 24–48 h, and the diameters of the inhibition zones were measured in millimeters and recorded as mean values (*n* = 5) to evaluate the antibacterial activity.

### In vitro evaluation of ZnO-NPs antimicrobial activity against *Pantoea sp*

Zinc oxide nanoparticles (ZnO-NPs; purity 99.9%) were obtained from Nano-Gate Company (Cairo, Egypt). The nanoparticles were spherical, with an average diameter of 30 ± 5 nm, and appeared as a white to light-yellow powder (molecular weight: 81.38 g/mol). ZnO-NPs were suspended in 10 mL of sterile deionized water and sonicated for 30 min using an ultrasonic processor (BANDELIN, Germany; 20 kHz) to minimize aggregation.

The antibacterial activity of ZnO-NPs against *Pantoea* sp. was evaluated using the agar well diffusion method [31]. Nutrient agar plates were prepared and inoculated using a spread plating method of *Pantoea* sp. Wells of 4 mm diameter were then punched into the agar, and 100 µL of ZnO-NP suspensions at concentrations of 0, 50, 100, and 200 mg L⁻^1^ were added [32]. Sterile distilled water served as the negative control. The plates were incubated at 28 °C for 24 h, and inhibition zones were measured in millimeters and recorded as mean values (*n* = 5) to evaluate antibacterial activity.

### Detection and identification of *Bacillus*-derived secondary metabolites using Gas Chromatography–Mass Spectrometry (GC–MS)

#### Extraction of bioactive secondary metabolites (SMs)

To analyze bioactive compounds, gas chromatography–mass spectrometry (GC–MS) was performed on the methanolic extract of the same *Bacillus* sp. used throughout this study for culture filtrate preparation and biocontrol assays. *Bacillus* sp. was grown in nutrient broth (NB) and incubated for 48 h at 27–29 °C. After incubation, the culture was centrifuged to separate the bacterial cells, and the supernatant containing extracellular metabolites was collected. The supernatant was evaporated to dryness using a rotary evaporator at 45 °C. The resulting residue was dissolved in 1 mL of methanol, filtered through a 0.2 μm syringe filter, and stored at 4 °C for 24 h prior to gas chromatography–mass spectrometry (GC–MS) analysis [33].

#### Profiling of secondary metabolites via GC–MS analysis

The chemical composition of a methanolic extract of *Bacillus* sp. was analyzed using GC-TSQ mass spectrometer (Thermo Scientific, Austin, TX, USA) equipped with a direct capillary column TG–5MS (30 m × 0.25 mm × 0.25 µm film thickness). The column oven temperature was initially held at 60 °C, and then increased at a rate of 5 °C/min to 250 °C, hold for 2 min, and further increased to 300 °C at a rate of 30 °C/min. The injector temperature was set at 270 °C. Helium was used as a carrier gas at a constant flow rate of 1 ml/min. A solvent delay was 4 min was applied, and 1 µl of diluted extract were injected automatically using an AS3000 autosampler in split mode. Electron ionization (EI) was conducted at 70 eV, and mass spectra were recorded in full scan mode over an *m/z* range of 50–650. The ion source and transfer line temperatures were set at 200 °C and 280 °C, respectively. Compounds were identified by comparing the obtained mass spectra with those in the WILEY (Wiley Registry of Mass Spectral Data, 9th Edition, Version 1.02) and NIST 14 (NIST/EPA/NIH) mass spectral databases, as described by Abd El-Kareem et al. [34].

### Experimental setup and treatments for *Pantoea* leaf spot management under greenhouse conditions

Healthy seeds of cucumber (*Cucumis sativus* L.), F1 hybrid cultivar ‘Happystar RZ’, were obtained from Rijk Zwaan (Netherlands) for use in the pot experiment. Seeds were surface-sterilized by immersion in 70% ethanol for 30 s, followed by agitation in a 2% sodium hypochlorite solution for 20 min. After rinsing three times with sterile distilled water, the seeds were placed in 90-mm glass Petri dishes lined with sterile, moistened filter paper and incubated in the dark at 28 °C for 36 h to germinate. Germinated seeds were sown in nursery cups filled with 300 g of sterilized nursery soil. After 30 days, seedlings at the 3–4 leaf stage were transplanted into pots (30 cm inner diameter) containing 10 kg of a sterilized clay:sand mixture (2:1,*v/v*). The experiment was conducted in a controlled greenhouse at the Plant Pathology Department, Faculty of Agriculture, Alexandria University, Egypt, under average day/night temperatures of 23/20 °C (± 2 °C), relative humidity of 76–80%, and a natural photoperiod of approximately 11/13 h light/dark [35].

Photosynthetically active radiation (PAR) was measured inside the glass greenhouse throughout the experimental period (November–January) using a PHOTOBIO LGBQM2 Advanced Quantum Sensor PAR Meter (Phantom PHOTOBIO, Ubuy Egypt). Daily PAR values ranged between 300 and 550 µmol photons m^−2^ s^−1^, with an average of approximately 400 µmol photons m^−2^ s^−1^ during peak daylight hours. These readings align with previously reported ranges for Mediterranean and Egyptian glass greenhouses during winter, where light transmissivity typically accounts for a 30–40% reduction in external PAR levels. Such values are well-documented to support optimal cucumber and other vegetable crop growth under comparable agro-climatic conditions [36, 37].

The experiment followed a randomized complete block design (RCBD) with five replicates per treatment. Sixteen treatment combinations were tested, comprising two levels of bacterial infection (inoculated with *Pantoea* sp. or non-inoculated), two levels of *Bacillus* culture filtrate (BCF) application (absence [–BCF] or presence [+ BCF]), and four concentrations of zinc oxide nanoparticles (ZnO-NPs) applied as foliar sprays (0, 50, 100, and 200 ppm).

Thirty-day-old cucumber seedlings were irrigated with a half-strength, zinc-free Hoagland and Arnon nutrient solution (pH 5.5–6.0), prepared according to Hewitt’s method [38]. Irrigation was applied via a surface drip system. Foliar ZnO-NP treatments were applied during the vegetative growth stage using a hand-held sprayer, with 200 mL of the respective solution applied per plant. The first application occurred one week after transplanting, followed by three subsequent applications at weekly intervals. Thus, the ZnO-NP treatments were applied four times in total: two applications before and two after pathogen inoculation (18 days after transplanting, DAT). Control plants were sprayed with autoclaved distilled water. To improve spray adhesion and leaf surface coverage, five drops of 80% Tween® 20 were added to each treatment solution [13].

*Bacillus* culture filtrate (BCF) was applied at two levels by foliar spraying to the point of runoff (approximately 200 mL per plant). The BCF was applied twice: 24 h before and 24 h after inoculation with *Pantoea* sp. Pathogen inoculation was performed 18 DAT, when cucumber plants had reached the 5–6 true leaf stage. A bacterial suspension of *Pantoea* sp. was injected into healthy cucumber leaves using a sterile syringe, while control plants were injected with sterile distilled water using the same method. All plants were maintained under greenhouse conditions and monitored daily. Disease symptoms, including foliar shriveling and necrosis, were assessed 15 days after inoculation. No symptoms developed in the control group.

### Measurements

#### Disease incidence and severity

Disease severity was assessed using a 1–7 scale adapted from Le et al. [39], where:

- 1= no symptoms,
- 2= a few necrotic spots on a few leaves,
- 3= a few necrotic spots on many leaves,
- 4= numerous spots with coalescence on a few leaves,
- 5= numerous coalesced spots on many leaves,
- 6= severe infection with leaf defoliation,
- 7= plant death.

This scale was applied to evaluate leaf spot disease progression, and severity ratings were recorded for infected plants in each replicate.

#### Vegetative growth parameters

At 75 days after transplanting (DAT), five cucumber plants were randomly selected from each treatment. Vegetative growth parameters, including plant height (cm), number of leaves per plant, and leaf area (dm^2^ per plant), were recorded. Leaf area was estimated using the method proposed by Blanco and Folegatti [40], which is specifically adapted for cucumber plants grown under greenhouse conditions.

#### Leaf nutrient composition

For mineral nutrient analysis, the most recently mature leaves were collected before the fruit set stage. Samples were oven-dried at 70 °C for 48 h and digested using appropriate acid digestion procedures. Total nitrogen (N) and potassium (K) contents were determined following the methods of Jones Jr [41]. Phosphorus (P) was measured using the vanadate–molybdate colorimetric method described by Page et al. [42]. Iron (Fe) and zinc (Zn) concentrations were also estimated according to Jones Jr [41].

#### Total phenolic content

Total phenolic content in cucumber leaves was determined using the Folin–Ciocalteu method, as described by Malik and Singh [43]. One gram of fresh leaf tissue was homogenized in 80% ethanol and centrifuged at 10,000 rpm for 30 min. The residue was re-extracted five times with 80% ethanol, and all supernatants were pooled and evaporated to near dryness. The remaining residue was dissolved in 5 mL of distilled water. A 0.2 mL aliquot of the extract was diluted to 3 mL with distilled water, followed by the addition of 0.5 mL Folin–Ciocalteu reagent. After 3 min, 20% Na_2_CO_3_ solution was added, and the mixture was immediately and thoroughly mixed. The tubes were incubated in boiling water for 1 min, and then cooled. Absorbance was measured at 650 nm using a spectrophotometer. Total phenolic content was expressed as mg gallic acid equivalent per gram of fresh weight (mg GAE g^−1^ F.W.).

#### Leaf chlorophyll content (SPAD Value)

Chlorophyll content was measured as SPAD values using portable chlorophyll meter (SPAD-502 Plus, Konica Minolta, Tokyo, Japan). Measurements were taken from the fifth fully expanded leaf from the apex on five randomly selected plants per treatment.

#### Fruit yield and quality

At the harvesting maturity stage, cucumber fruits were randomly collected from each treatment to evaluate yield and quality parameters. The measured parameters included fruit length (cm), fruit diameter (cm), average fruit weight (g fruit^−1^), number of fruits per plant, and total yield (kg plant^−1^). Fruit diameter was determined using a digital caliper (GELIXI, Hangzhou, China) by gently measuring the equatorial diameter at the widest point of each fruit. All measurements were taken with precision and care to avoid causing any physical damage to the fruit surface or epidermal tissues.

Fruit quality parameters—including total soluble solids (TSS, °Brix), ascorbic acid (vitamin C), pH, titratable acidity, total carotenoids, and cucurbitacins—were analyzed following standard procedures according to the methodology described by AOAC [44].

Total soluble solids (TSS, °Brix) were determined in freshly extracted cucumber juice using a digital refractometer (SM-100, EasyTest, Egypt). Approximately 2 mL of juice was placed on the prism, and readings were recorded at room temperature. The prism surface was rinsed with distilled water and wiped dry between samples to avoid cross-contamination.

Vitamin C (ascorbic acid) content was determined spectrophotometrically following AOAC [44], using a UV–Visible spectrophotometer (UV-1280, Shimadzu, Japan). Juice extracted from 10 g of fresh fruit was reacted with 2,6-dichlorophenolindophenol reagent, and absorbance was measured at 530 nm. Vitamin C concentrations were quantified using an external calibration curve prepared from L-ascorbic acid standards, and the results were expressed as mg 100 g^−1^ fresh weight (FW). To verify accuracy, iodometric titration was performed on randomly selected samples.

pH was measured directly in 10 mL of clarified cucumber juice using a portable pH meter (HI98103, HANNA Instruments, Egypt) that was calibrated with pH 4.0 and 7.0 buffer standards prior to each measurement batch.

Titratable acidity was determined by titrating 10 mL of cucumber juice with 0.1 N NaOH using phenolphthalein as an indicator, following AOAC [44]. The results were expressed as a percentage of citric acid (% CA; equivalent to g 100 g^−1^ FW).

Total carotenoids were extracted by homogenizing fruit tissue in a chloroform:methanol mixture (2:1, *v*/*v*), following the method of Mozafari et al. [45]. The extract was filtered, evaporated under a nitrogen stream, and analyzed using UHPLC (Shimadzu, Japan) equipped with a C32 column and a photodiode array detector set at 450 nm. Quantification was carried out using β-carotene as a reference standard, and the results were expressed as mg g^−1^ fresh weight (FW).

Cucurbitacins were quantified using HPLC (Agilent 1260 Infinity II, USA) equipped with a C18 column and a UV detector set at 230 nm, following the method of Chanda et al. [46]. Methanolic extracts were filtered, evaporated to dryness, reconstituted in the mobile phase, and analyzed using cucurbitacin B and E (Sigma-Aldrich) as external reference standards. The results were expressed as mg kg^−1^dry weight (DW).

### Statistical analysis

The in vitro and greenhouse experiments were each conducted twice. For each experiment, data were subjected to analysis of variance (ANOVA), and a combined ANOVA was performed across repetitions due to homogeneity of error variances. The greenhouse trials were conducted during two consecutive seasons (2022/2023 and 2023/2024) to confirm the reproducibility of results. In both seasons, seedlings were transplanted on 1 November, and the experiments concluded by the end of January. All statistical analyses were performed using CoStat software [47], (version 6.400; CoHort Software, Monterey, CA, USA). ANOVA was conducted following the procedures of Gomez and Gomez [48]. Mean comparisons were carried out using the least significant difference (LSD) test at probability levels of *p* ≤ 0.05 (*) and *p* ≤ 0.01 (**).

## Results

### Phylogenetic analyses of *Pantoea* sp. and *Bacillus* sp. Isolates

**Fig. 4:**
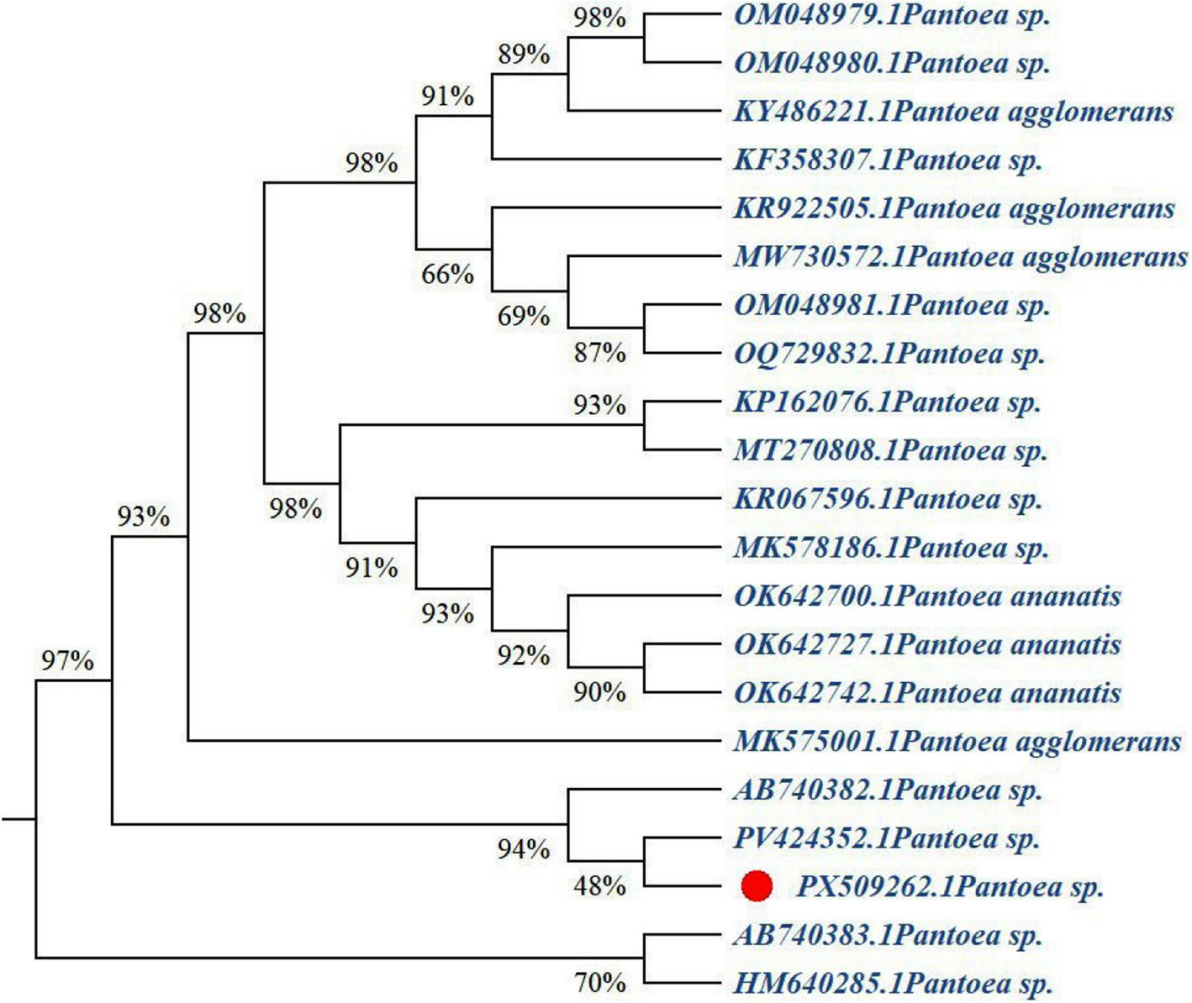
Phylogenetic tree constructed using the neighbor-joining method in MEGA 11 software, showing the evolutionary relationships among various *pantoea* isolates based on gene sequence data. The studied isolate (PX509262.1, *Pantoea* sp., indicated by the red dot) clusters with other *Pantoea* species, with similar percentages displayed on the branches. The tree highlights the genetic relatedness and diversity among the analyzed *Pantoea* isolates

The phylogenetic tree was constructed using MEGA 11 software and the Neighbor-Joining method to illustrate the evolutionary relationships among various *Pantoea* isolates (Fig. 4). The studied isolate (PX509262.1*, Pantoea* sp*.)* is positioned within a cluster alongside other *Pantoea* species and strains. The bootstrap values shown along the tree branches, which range from 48 to 98%, indicate varying degrees of genetic relatedness between PX509262.1 and the *Pantoea* isolates included in the analysis. These results demonstrate the overall genetic diversity among the *Pantoea* isolates and support the molecular identification of PX509262.1 as a member of the *Pantoea* genus (Fig. 4).

**Fig. 5:**
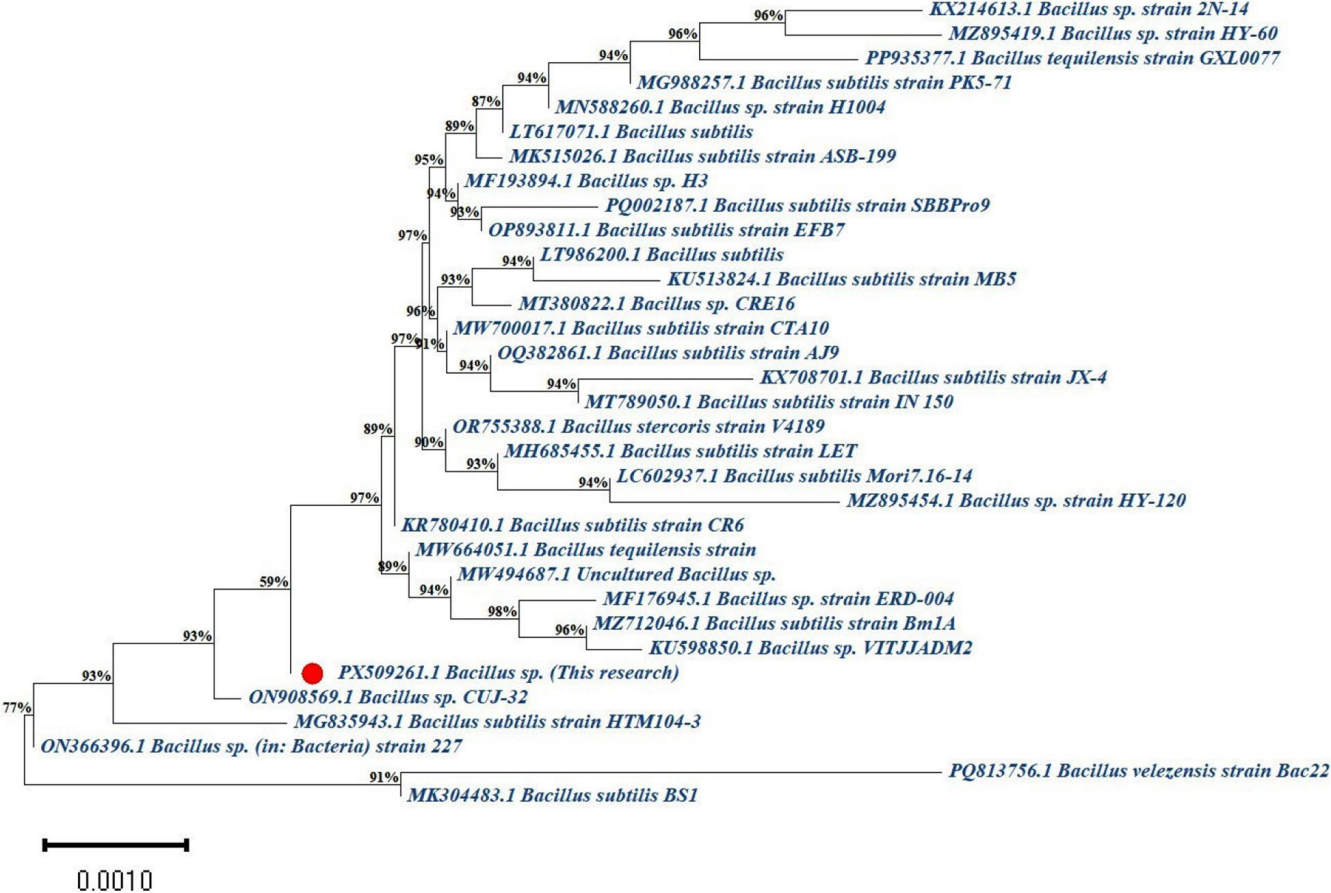
Phylogenetic tree constructed using the Neighbor-Joining method in MEGA 11 software, showing the evolutionary relationships among various *Bacillus* isolates based on gene sequence data. The studied isolate (PX509261.1 *Bacillus* sp., indicated by the red dot) clusters with other *Bacillus* species, with similar percentages displayed on the branches. The tree highlights the genetic relatedness and diversity among the analyzed *Bacillus* isolates

The phylogenetic tree was constructed using MEGA 11 software and the Neighbor-Joining method to illustrate the evolutionary relationships among various *Bacillus* isolates. As shown in Fig. 5, the studied isolate (PX509261.1 *Bacillus* sp.) is positioned within a cluster together with other *Bacillus* species. The similarity percentages shown along the tree branches, which range from 59 to 98%, indicate varying degrees of genetic relatedness between PX509261.1 and the *Bacillus* isolates included in the analysis. These results demonstrate the overall genetic diversity among the *Bacillus* isolates and support the molecular identification of PX509261.1 as a member of the *Bacillus* genus.

### In vitro antagonistic effect of *Bacillus* culture filtrate against *Pantoea* sp

**Fig. 6:**
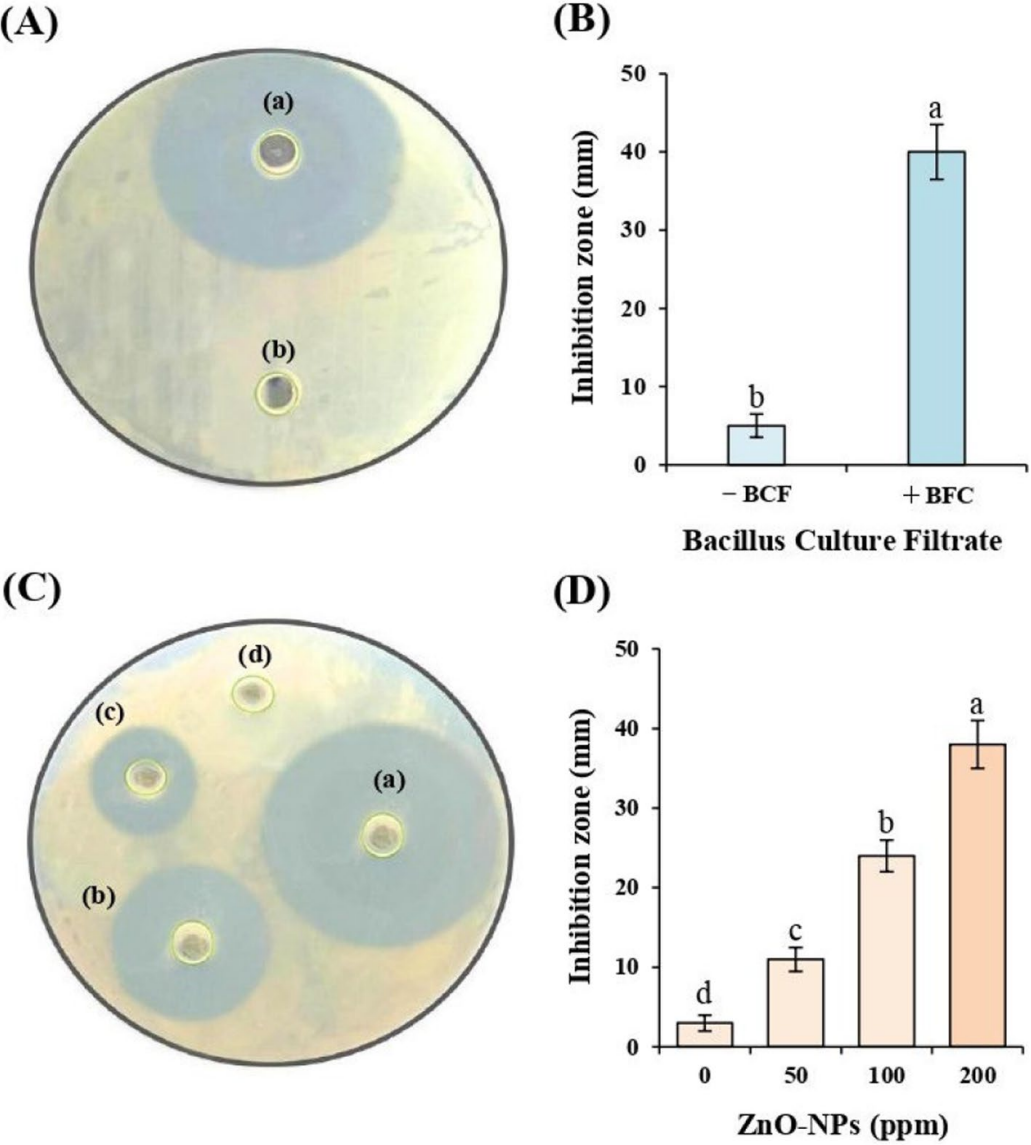
Antibacterial activity of *Bacillus* culture filtrate (BCF) and ZnO-NPs against *Pantoea* sp. (**A**–**B**) Inhibitory effect of BCF (a) compared to the control (b).(**C**–**D**) Inhibition zones produced by ZnO-NPs at concentrations of 50 ppm (c), 100 ppm (b), and 200 ppm (a), compared to the control (d). Bar graphs represent the corresponding inhibition zone diameters (mm), presented as mean ± standard deviation (SD) of five replicates (*n* = 5). Different letters above the bars denote statistically significant differences according to LSD test at *p* ≤ 0.01 (**)

The culture filtrate of *Bacillus* sp. (BCF) exhibited potent antibacterial activity against *Pantoea* sp. (Fig. 6A–B). Application of BCF produced a significantly larger inhibition zone (40.0 ± 3.5 mm) compared to the untreated control (5.0 ± 1.5 mm). The difference was statistically significant (*p* ≤ 0.01), with the LSD test indicating distinct groups as denoted by different letters on the corresponding bar graph (Fig. 6B).

### In vitro antimicrobial activity of ZnO-NPs against *Pantoea* sp

As shown in Fig. 6C–D, the inhibition zone diameter increased significantly (*p* ≤ 0.01) with increasing ZnO-NPs concentrations. The control (0 ppm) exhibited minimal inhibition (3.0 ± 1.0 mm), whereas 50, 100, and 200 ppm ZnO-NPs resulted in inhibition zones of 11.0 ± 1.5 mm, 24.0 ± 2.0 mm, and 38.0 ± 3.0 mm, respectively. The antimicrobial activity of 200 ppm ZnO-NPs was significantly higher than all other concentrations, as confirmed by LSD post hoc comparisons.

### GC–MS-based metabolite profiling of *Bacillus* culture filtrate

The GC–MS analysis of the methanolic extract of *Bacillus* sp. identified twenty major chemical constituents with ≥ 65% match to the Wiley Registry® of Mass Spectral Data (Table 2). These compounds varied in retention time (RT) from 4.31 to 24.40 min and included fatty acids, esters, hydrocarbons, and nitrogen-containing molecules. The most abundant compounds, based on area percentage, were oleic acid (45.96%), cis-13-octadecenoic acid (40.46%), n-hexadecanoic acid (35.63%), 10-octadecenoic acid, methyl ester (21.50%), and hexadecanoic acid, methyl ester (15.89%).

**Table 2:** Major chemical compounds identified in the methanolic extract of *Bacillus* sp. using GC–MS, with ≥ 65% match quality to the Wiley Registry® of Mass Spectral Data, and their associated biological activities

Serial No	Compound Name	RT (min)	Area (%)	Molecular formula	Cas #	Biological Properties	References
1	7,7,9,9,11,11-Hexamethyl-3,6,8,10,12,15-hexaoxa-7,9,11-trisilaheptadecane	4.31	1.62	C_14_H_36_O_6_Si_3_	N/A	Antimicrobial activity	[49]
2	Cyclotrisiloxane, hexamethyl-	4.35	5.74	C_6_H_18_O_3_Si_3_	541–05–9	Antioxidant; antimicrobial activities	[49, 50]
3	2,5,5-trimethyl-2-(butylthio)cycloheptatriene	5.19	5.40	C_14_H_22_S	N/A	Antibacterial activity	[51]
4	2-thiopheneethanol, 5-(4,5-dihydro-4,4-dimethyl-2-oxazolyl)-	5.19	1.94	C_11_H_15_NO_2_S	97759–85-8	Antibacterial activities and antioxidant properties	[52]
5	6,7-Dimethoxy-2-methyl-3,4-dihydro[1-D] isoquinolinium ion	5.19	1.94	C_12_H_15_DNO_2_	N/A	Cytotoxic; antioxidant; antibiotic activities	[53]
6	Hexadecanoic acid, methyl ester	20.46	15.89	C_17_H_34_O_2_	112–39-0	Antibacterial activities and antioxidant properties	[52]
7	Pentadecanoic acid, 14-methyl-,methyl ester	20.46	7.87	C_17_H_34_O_2_	5129–60-2	Antifungal; antimicrobial activities	[54]
8	n-Hexadecanoic acid or palmitic acid	21.41	35.63	C_16_H_32_O_2_	57–10-3	Plant growth promoter; antioxidant; antibacterial activities	[55–57]
9	7,10-Octadecadienoic acid, methyl ester	23.07	10.24	C_19_H_34_O_2_	56554–24-6	Antibacterial; antioxidant; antimicrobial activities	[58]
10	9,12-Octadecadienoic acid, methyl ester, (E,E)-	23.07	10.44	C_19_H_34_O_2_	2566–97-4	Antioxidant; antifungal; antibacterial activities	[59]
11	8,11-Octadecadienoic acid, methyl ester	23.07	5.24	C_19_H_34_O_2_	56599–58-7	Antibacterial; antioxidant; antimicrobial activities	[58]
12	8-octadecenoic acid, methyl ester	23.16	8.59	C19H36O2	2345–29-1	Antioxidant; antimicrobial activities	[60]
13	9-Octadecenoic acid, methyl ester, (E)-	23.16	8.59	C_19_H_36_O_2_	1937–62-8	Cytotoxic; antioxidant; antiproliferative; antimicrobial; free radical-scavenging activities	[61]
14	10-Octadecenoic acid, methyl ester	23.27	21.50	C_19_H_36_O_2_	13481–95-3	Antibacterial; antioxidant; antimicrobial activities	[58, 62]
15	16-octadecenoic acid, methyl ester	23.27	4.5	C_19_H_36_O_2_	56554–49-5	Antibacterial; antioxidant; antimicrobial; antiviral activities	[63]
16	Octadecanoic acid, methyl ester	23.58	5.22	C_19_H_38_O_2_	112–61-8	Antibacterial; antioxidant; antimicrobial activities	[58]
17	Methyl-9,9,10,10-D4-octadecanoate	23.58	2.22	C_19_H_34_D_4_O_2_	56554–85-9	Potential antimicrobial and antiproliferative activities	[64]
18	Oleic Acid	24.11	45.96	C_18_H_34_O_2_	112–80-1	Antibacterial; antioxidant; antimicrobial; plant growth-promoting activities	[59, 64]
19	cis-13-Octadecenoic acid	24.40	40.46	C_18_H_34_O_2_	13126–39-1	Antibacterial; antioxidant; antimicrobial activities	[58]
20	Octadecanoic Acid or stearic acid	24.40	5.63	C_18_H_36_O_2_	57–11-4	Antiviral; antibacterial; cytotoxic; antioxidant activities	[65]

^*^RT  Retention time, *N/A* Not available, *Cas* Chemical abstracts service, *Cas #* Cas registry number

Other notable compounds present in lower concentrations included 9,12-octadecadienoic acid, methyl ester (E,E)- (10.44%), 7,10-octadecadienoic acid, methyl ester (10.24%), 8-octadecenoic acid, methyl ester (8.59%), 9-octadecenoic acid, methyl ester (E)- (8.59%), and pentadecanoic acid, 14-methyl-, methyl ester (7.87%). Several minor compounds such as cyclotrisiloxane, hexamethyl-; methyl-9,9,10,10-D4-octadecanoate; and 2,5,5-trimethyl-2-(butylthio)cycloheptanone were also detected, each comprising less than 6% of the total area.

According to our findings, and supported by numerous studies summarized in Table 2, the antagonistic *Bacillus* sp. is capable of producing a diverse array of bioactive chemical compounds with demonstrated antibacterial, antioxidant, and plant growth-promoting activities.

### Effect of treatments on *Pantoea* leaf spot disease under greenhouse conditions

#### Bacterial spot severity

**Fig. 7:**
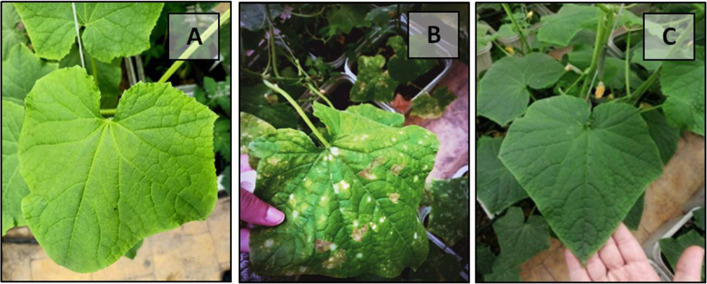
Cucumber plant leaves at 45 DAT under different treatments. **A** Healthy control leaf without inoculation, (**B**) Leaf spot symptoms following artificial inoculation with *Pantoea* sp., and (**C**) Suppression of *Pantoea* leaf spot symptoms in plants treated with 200 ppm ZnO-NPs and BCF

The severity of bacterial leaf spot (BLS) symptoms caused by *Pantoea* sp. in cucumber leaves was significantly affected (*p* ≤ 0.01) by foliar application of *Bacillus* culture filtrate (BCF), zinc oxide nanoparticles (ZnO-NPs), and their combinations (Table 3; Fig. 7). In the absence of BCF, disease severity decreased progressively with increasing ZnO-NPs concentrations from 0 to 200 ppm, with a marked reduction from 5.0 at 0 ppm to 2.0 at 200 ppm. The co-application of BCF significantly enhanced disease suppression at each ZnO-NPs level. The most pronounced reduction was observed in plants treated with 200 ppm ZnO-NPs + BCF, which recorded the lowest severity score of 1.1.

**Table 3:** Effect of *Bacillus* culture filtrate (BCF) and zinc oxide nanoparticles (ZnO-NPs) on bacterial spot severity in cucumber plants infected with *Pantoea* sp

**ZnO-NPs** **(ppm)**	*Mean Bacterial Spot Severity		**Mean**
	***Bacillus* culture filtrate (BCF)**^¶^		
	**- BCF**	** + BCF**	
0	5.0 ± 0.14a	3.5 ± 0.12c	4.3 ± 0.80a
50	4.4 ± 0.13b	2.2 ± 0.11e	3.3 ± 1.14b
100	2.9 ± 0.10d	1.5 ± 0.15 g	2.2 ± 0.74c
200	2.0 ± 0.11f	1.1 ± 0.14 h	1.5 ± 0.47d
Mean	3.6 ± 1.23a	2.1 ± 0.94b	
L.S.D. _0.01_	ZnO-NPs = 0.1425	BCF = 0.1008	ZnO-NPs x BCF = 0.2016
F-value	ZnO-NPs = 1080.3**	BCF = 1635.4**	ZnO-NPs x BCF = 53.7**
C.V	4.09%		

^¶^BCF: *Bacillus* culture filtrate, where (–BCF) indicates absence and (+ BCF) indicates presence. Values are expressed as mean ± SD (*n* = 5). Means followed by the same alphabetical letter(s) in common are not significantly different at* p* ≤ 0.01 (**). C.V., coefficient of variation (%)

^*^Disease severity was rated on a 1–7 scale: 1 = symptomless, 2 = a few necrotic spots on a few leaves, 3 = a few necrotic spots on many leaves, 4 = many spots with coalescence on few leaves, 5 = many spots with coalescence on many leaves, 6 = severe disease and leaf defoliation, and 7 = plant dead

Visual assessments supported these quantitative findings (Fig. 7). Healthy, uninoculated plants exhibited green, symptom-free leaves (Fig. 7A), while inoculated, untreated plants developed typical necrotic spots (Fig. 7B). In contrast, plants receiving the combined treatment of ZnO-NPs at 200 ppm with BCF showed nearly complete suppression of BLS symptoms (Fig. 7C).

#### Vegetative growth parameters

Vegetative growth traits of cucumber plants, assessed at 75 DAT, were significantly affected by *Bacillus* culture filtrate (BCF) and zinc oxide nanoparticles (ZnO-NPs), whether applied alone and in combination, and in the presence or absence of *Pantoea* sp. infection (Table 4).

**Table 4:** Vegetative growth parameters of cucumber plants at 75 days after transplanting (DAT), as influenced by foliar application of *Bacillus* culture filtrate (BCF) and zinc oxide nanoparticles (ZnO-NPs), with or without *Pantoea* sp. infection

BLS Infection	BCF Treatment	ZnO-NPs (ppm)	Plant height (cm)	No. of leaves/plant	Leaf area (dm^2^ per plant)
Without	− BCF	0	121 ± 3.1i	20 ± 0.71i	66 ± 1.00j
		50	135 ± 3.9 g	21 ± 0.45 h	72 ± 1.14 h
		100	166 ± 5.2d	27 ± 0.55e	82 ± 1.10d
		200	193 ± 3.6c	35 ± 0.84b	92 ± 1.58b
	+ BCF	0	149 ± 3.7f	22 ± 0.55 g	70 ± 1.48 h
		50	168 ± 6.3d	25 ± 0.71f	76 ± 1.48f
		100	211 ± 5.8b	29 ± 1.00d	85 ± 1.79c
		200	251 ± 6.8a	39 ± 0.89a	99 ± 1.95a
With	− BCF	0	84 ± 3.6 k	18 ± 0.55j	57 ± 0.71 l
		50	110 ± 4.0j	20 ± 0.84i	68 ± 0.84i
		100	128 ± 2.6 h	25 ± 0.55f	73 ± 0.45 g
		200	146 ± 3.6f	30 ± 0.84d	84 ± 1.00c
	+ BCF	0	116 ± 4.7i	21 ± 0.71 h	64 ± 1.14 k
		50	129 ± 5.4 h	24 ± 0.55f	71 ± 1.41 h
		100	155 ± 4.2e	28 ± 0.71e	78 ± 0.71e
		200	210 ± 4.1b	33 ± 0.45c	91 ± 0.89b

*BLS* bacterial leaf spot caused by *Pantoea* sp., *BCF* *Bacillus* culture filtrate, where (–BCF) indicates absence and (+ BCF) indicates presence. Values represent as mean ± SD (*n* = 5). Means within each column followed by the same alphabetical letter(s) do not differ significantly at *p* ≤ 0.05(*)

Under non-infected conditions, foliar application of ZnO-NPs resulted in dose-dependent increases in plant growth. In the absence of BCF, increasing ZnO-NPs concentrations from 0 to 200 ppm resulted in significant increases in plant height, from 121 ± 3.1 cm to 193 ± 3.6 cm (*p* ≤ 0.05). When combined with BCF, further improvements were observed, with plant height reaching 251 ± 6.8 cm at 200 ppm ZnO-NPs, which was significantly higher than all other treatments (*p* ≤ 0.05). Parallel increases were recorded for the number of leaves per plant (from 20.0 ± 0.71 to 39.0 ± 0.89) and leaf area (from 66 ± 1.00 to 99 ± 1.95 dm^2^), indicating a strong synergistic effect between BCF and ZnO-NPs in promoting vegetative growth.

Infection with *Pantoea* sp. markedly reduced growth, as untreated infected plants exhibited the lowest values across all parameters (84 ± 3.6 cm height, 18.0 ± 0.55 number of leaves per plant, and 57 ± 0.71 dm^2^ leaf area). However, the application of ZnO-NPs, with or without BCF, mitigated these adverse effects. Notably, the co-application of BCF and 200 ppm ZnO-NPs significantly restored growth performance, with plant height, number of leaves per plant, and leaf area reaching 210 ± 4.1 cm, 33 ± 0.45, and 91 ± 0.89 dm^2^, respectively.

Overall, the combined treatment of BCF and ZnO-NPs significantly promoted vegetative growth in cucumber under both healthy and pathogen-stressed conditions. The statistically significant improvements observed across treatments (*p* ≤ 0.05) confirm the synergistic role of these agents in promoting cucumber growth and alleviating the negative impact of bacterial leaf spot caused by *Pantoea* sp.

#### Leaf nutrient composition

The foliar application of *Bacillus* culture filtrate (BCF), zinc oxide nanoparticles (ZnO-NPs), and their combinations significantly influenced the nutrient composition (N, P, K, Zn, and Fe) of cucumber leaves under both infected and non-infected conditions (Table 5).

**Table 5:** Leaf chemical composition of cucumber plants in response to foliar application of *Bacillus* culture filtrate (BCF) and zinc oxide nanoparticles (ZnO-NPs), with or without *Pantoea* sp. infection

BLS Infection	BCF Treatment	ZnO-NPs (ppm)	Leaf Chemical Composition				
			**(%)**			**(μg/g), of D.W**	
			**N**	**P**	**K**	**Zn**	**Fe**
Without	- BCF	0	2.9 ± 0.04 h	0.27 ± 0.01i	2.4 ± 0.04 k	13 ± 0.45 k	49 ± 0.71j
		50	3.3 ± 0.07 g	0.34 ± 0.02gh	2.9 ± 0.08i	23 ± 0.45 h	59 ± 1.67 h
		100	4.0 ± 0.09d	0.36 ± 0.01f	3.2 ± 0.07 fg	38 ± 0.89e	69 ± 1.48f
		200	4.5 ± 0.07b	0.43 ± 0.03c	3.6 ± 0.05d	55 ± 0.71b	82 ± 1.67d
	+ BCF	0	3.8 ± 0.08ef	0.38 ± 0.02ef	3.1 ± 0.07gh	19 ± 0.45i	64 ± 1.67 g
		50	4.1 ± 0.10d	0.40 ± 0.01de	3.4 ± 0.07e	27 ± 0.55 g	74 ± 2.61e
		100	4.7 ± 0.07b	0.47 ± 0.01b	3.8 ± 0.08c	48 ± 0.84c	85 ± 1.67c
		200	5.1 ± 0.06a	0.52 ± 0.03a	4.5 ± 0.10a	62 ± 0.71a	99 ± 2.68a
With	- BCF	0	2.6 ± 0.04i	0.18 ± 0.02j	1.7 ± 0.04 m	10 ± 0.89 l	39 ± 0.71 k
		50	2.9 ± 0.09 h	0.27 ± 0.01i	2.3 ± 0.08 l	20 ± 0.55i	48 ± 1.67j
		100	3.3 ± 0.11 g	0.32 ± 0.01 h	2.7 ± 0.07j	30 ± 0.71f	55 ± 1.48i
		200	3.8 ± 0.07e	0.37 ± 0.02f	3.2 ± 0.04 fg	47 ± 0.89c	69 ± 1.67f
	+ BCF	0	3.3 ± 0.10 g	0.33 ± 0.01 h	2.6 ± 0.07j	15 ± 0.55j	54 ± 1.67i
		50	3.7 ± 0.08f	0.36 ± 0.02 fg	3.0 ± 0.05hi	24 ± 0.71 h	59 ± 1.67 h
		100	4.0 ± 0.09d	0.42 ± 0.01 cd	3.3 ± 0.09f	43 ± 0.89d	76 ± 2.61e
		200	4.3 ± 0.07c	0.45 ± 0.03bc	4.1 ± 0.08b	54 ± 0.84b	89 ± 2.83b

*BLS* bacterial leaf spot caused by *Pantoea* sp., *BCF* *Bacillus* culture filtrate, where (–BCF) indicates absence and (+ BCF) indicates presence. Values represent as mean ± SD (*n* = 5). Means within each column followed by the same alphabetical letter(s) do not differ significantly at *p* ≤ 0.01 (**)

Under non-infected conditions, untreated plants showed the lowest levels of nitrogen (2.9%), phosphorus (0.27%), and potassium (2.4%). Foliar application of ZnO-NPs alone led to a dose-dependent enhancement in nutrient accumulation. At 200 ppm ZnO-NPs, leaf N, P, and K contents increased to 4.5%, 0.43%, and 3.6%, respectively. The co-application of BCF further boosted nutrient uptake. The highest nutrient levels were recorded in the BCF and 200 ppm ZnO-NPs treatment, where N reached 5.1%, P reached 0.52%, and K increased to 4.5%. These values were statistically higher (*p* ≤ 0.01) than all other treatments.

In the presence of *Pantoea* sp. infection, nutrient content was generally reduced in untreated plants, with N at 2.6%, P at 0.18%, and K at 1.7%. ZnO-NPs application under infected conditions significantly improved these levels. A dose-dependent increase was observed, especially when combined with BCF. The BCF and 200 ppm ZnO-NPs treatment yielded the highest nutrient concentrations under infection, reaching 4.3% N, 0.45% P, and 4.1% K.

Regarding micronutrients, zinc and iron contents were significantly enhanced by ZnO-NPs application. In non-infected plants, leaf Zn increased from 13 μg/g in untreated control to 55 μg/g with 200 ppm ZnO-NPs, and to 62 μg/g when combined with BCF. Similarly, Fe content increased from 49 μg/g to 82 μg/g and 99 μg/g under the same respective treatments. In infected plants, Zn and Fe contents followed a comparable trend, with maximum values (54 μg/g and 89 μg/g, respectively) observed under the BCF and 200 ppm ZnO-NPs treatment. All these increases were statistically significant (*p* ≤ 0.01) compared to controls.

These results demonstrate the positive interaction between BCF and ZnO-NPs in enhancing both macro- and micronutrient content of cucumber leaves, especially under biotic stress conditions.

#### Total phenolic content

**Fig. 8:**
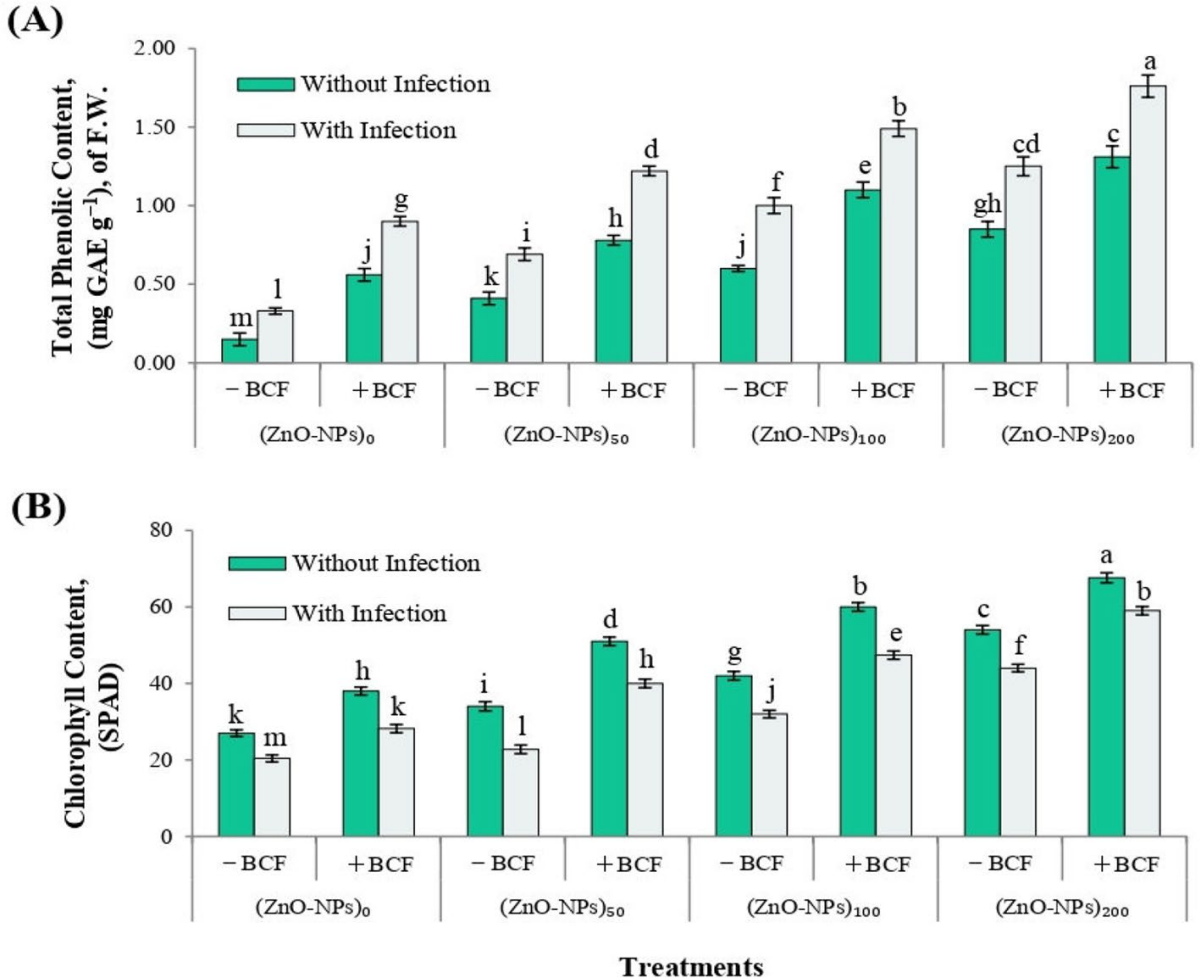
Effects of *Bacillus* culture filtrate (BCF) and zinc oxide nanoparticles (ZnO-NPs) on (**A**) total phenolic content (mg GAE g⁻.^1^ fresh weight) and (**B**) chlorophyll content (SPAD units) in cucumber leaves under *Pantoea* leaf spot infection and non-infection conditions. Treatments included foliar application of ZnO-NPs (0, 50, 100, and 200 ppm) with or without BCF. Bars represent mean ± standard deviation (SD) of five replicates (*n* = 5). Different letters above bars indicate statistically significant differences at *p* ≤ 0.01 according to LSD test (**)

The total phenolic content in cucumber leaves was significantly influenced by foliar application of *Bacillus* culture filtrate (BCF), zinc oxide nanoparticles (ZnO-NPs), and their combinations, under both infected and non-infected conditions (Fig. 8A). Plants grown under pathogen-free conditions and receiving no treatment exhibited the lowest phenolic levels (0.15 mg GAE g^−1^ FW). In contrast, *Pantoea* sp. infection alone induced an increase in phenolic content to 0.33 mg GAE g^−1^ FW.

Application of ZnO-NPs alone under non-infected conditions induced a concentration-dependent rise in phenolic content, from (0.41 mg GAE g^−1^ FW) at 50 ppm to (0.85 mg GAE g^−1^ FW) at 200 ppm. When BCF was co-applied, a pronounced enhancement was observed at all nanoparticle concentrations. The maximum accumulation of phenolics (1.31 mg GAE g^−1^ FW) was recorded in the combination of BCF and 200 ppm ZnO-NPs under non-infected conditions.

Similarly, under infected conditions, the synergistic treatments significantly boosted phenolic content compared to the infected control (0.33 mg GAE g^−1^ FW). The BCF and 200 ppm ZnO-NPs treatment resulted in the highest phenolic content (1.76 mg GAE g^−1^ FW). Incremental ZnO-NPs concentrations showed a dose-dependent enhancement in phenolic content, especially when combined with BCF. Statistical analysis confirmed significant differences (*p* ≤ 0.01) among treatments, confirming the positive interaction between BCF and ZnO-NPs in inducing phenolic biosynthesis.

#### Leaf chlorophyll content (SPAD value)

Chlorophyll content, as indicated by SPAD values, was significantly influenced by foliar application of *Bacillus* culture filtrate (BCF), zinc oxide nanoparticles (ZnO-NPs), and their combined treatments, under both infected and non-infected conditions (Fig. 8B). The lowest SPAD reading (20.4) was recorded in infected plants without any treatment, indicating severe chlorosis caused by *Pantoea* sp. infection. Similarly untreated but non-infected plants also exhibited low chlorophyll content (27.0).

Under non-infected conditions, a dose-dependent increase in SPAD values was observed with ZnO-NPs treatments. Application of 50 ppm ZnO-NPs raised SPAD to 34.0, which further increased to 42.0 at 100 ppm and reached 54.0 at 200 ppm. Co-application with BCF significantly enhanced chlorophyll content at all levels. The combination of BCF with 200 ppm ZnO-NPs resulted in the highest SPAD value of all treatments.

In infected plants, a similar trend was noted. Chlorophyll content improved progressively with increasing ZnO-NPs concentrations, from 22.8 (50 ppm) to 32.0 (100 ppm) and 44.0 (200 ppm). BCF enhanced the effectiveness of each dose, with the highest value (59.0) observed in plants treated with BCF + 200 ppm ZnO-NPs. This treatment successfully restored chlorophyll levels in infected plants to levels comparable to or higher than untreated healthy plants.

#### Fruit yield and quality parameters

Fruit yield and quality traits of cucumber plants were significantly influenced by foliar application of *Bacillus* culture filtrate (BCF) and zinc oxide nanoparticles (ZnO-NPs), both individually and in combination, under both infected and non-infected conditions (Table 6).

**Table 6:** Fruit yield parameters of cucumber plants at harvesting maturity as affected by foliar application of *Bacillus* culture filtrate (BCF) and zinc oxide nanoparticles (ZnO-NPs), with or without *Pantoea sp.* infection

BLS Infection	BCF Treatment	ZnO-NPs (ppm)	Fruit length (cm)	Fruit diameter (cm)	Average fruit weight (g Fruit^−1^)	Number of fruits/plant	Total yield (kg plant^−1^)
Without	- BCF	0	9.8 ± 0.27i	2.5 ± 0.05j	98 ± 1.4i	6.8 ± 0.45i	0.86 ± 0.05j
		50	11.8 ± 0.45 h	3.4 ± 0.11f	107 ± 1.5f	9.0 ± 0.71 fg	1.62 ± 0.11 g
		100	13.6 ± 0.55f	3.6 ± 0.04e	112 ± 1.9d	11.2 ± 0.45d	2.54 ± 0.09d
		200	17.8 ± 0.45b	4.2 ± 0.07b	123 ± 1.6a	14.4 ± 0.55b	3.06 ± 0.09b
	+ BCF	0	12.6 ± 0.55 g	3.0 ± 0.08 h	102 ± 1.1gh	8.0 ± 0.71 h	1.14 ± 0.09i
		50	15.4 ± 0.55d	3.6 ± 0.04e	109 ± 1.5e	10.2 ± 0.45e	2.06 ± 0.11f
		100	15.2 ± 0.45d	3.8 ± 0.08d	114 ± 1.8 cd	12.0 ± 0.71c	2.70 ± 0.10c
		200	19.6 ± 0.89a	4.8 ± 0.10a	125 ± 1.9a	16.2 ± 0.45a	3.38 ± 0.08a
With	- BCF	0	7.7 ± 0.27j	2.0 ± 0.08 k	88 ± 1.0 k	3.4 ± 0.55 k	0.34 ± 0.05 k
		50	9.6 ± 0.42i	2.5 ± 0.04j	90 ± 1.5 k	4.6 ± 0.55j	0.92 ± 0.08j
		100	12.2 ± 0.27gh	3.3 ± 0.05 g	100 ± 1.6 h	8.4 ± 0.55gh	1.30 ± 0.05 h
		200	14.5 ± 0.35e	3.6 ± 0.05e	115 ± 1.1c	10.2 ± 0.84e	2.12 ± 0.08f
	+ BCF	0	10.2 ± 0.45i	2.9 ± 0.04i	94 ± 1.1j	5.2 ± 0.45j	0.82 ± 0.08j
		50	12.8 ± 0.45 g	3.5 ± 0.07e	103 ± 1.5 g	7.2 ± 0.45i	1.56 ± 0.09 g
		100	15.4 ± 0.55d	3.4 ± 0.05f	109 ± 1.7e	9.4 ± 0.55f	2.06 ± 0.05f
		200	17.0 ± 0.71c	3.9 ± 0.07c	119 ± 1.8b	14.2 ± 0.45b	2.42 ± 0.08e

*BLS* bacterial leaf spot caused by *Pantoea* sp., *BCF* *Bacillus* culture filtrate, where (–BCF) indicates absence and (+ BCF) indicates presence. Values represent as mean ± SD (*n* = 5). Means within each column followed by the same alphabetical letter(s) do not differ significantly at *p* ≤ 0.05(*)

Under non-infected conditions, ZnO-NPs application led to a dose-dependent increase in all measured parameters. In the absence of BCF, increasing ZnO-NPs concentrations from 0 to 200 ppm significantly enhanced fruit length from 9.8 ± 0.27 cm to 17.8 ± 0.45 cm, and fruit diameter from 2.5 ± 0.05 cm to 4.2 ± 0.07 cm (*p* ≤ 0.05). Average fruit weight increased from 98 ± 1.4 g to 123 ± 1.6 g, and total yield rose from 0.86 ± 0.05 kg to 3.06 ± 0.09 kg. When combined with BCF, further enhancements were recorded, with the highest values observed at 200 ppm ZnO-NPs: fruit length (19.6 ± 0.89 cm), fruit diameter (4.8 ± 0.10 cm), average fruit weight (125 ± 1.9 g), number of fruits per plant (16.2 ± 0.45), and total yield (3.38 ± 0.08 kg), all significantly higher than the untreated control and all other treatments (*p* ≤ 0.05).

Under *Pantoea* sp. infection, fruit yield and quality were substantially reduced in untreated plants, with the lowest fruit length (7.7 ± 0.27 cm), fruit diameter (2.0 ± 0.08 cm), average fruit weight (88 ± 1.0 g), and total yield (0.34 ± 0.05 kg/plant). Application of ZnO-NPs alone partially mitigated the negative effects of infection, while the combination of BCF and ZnO-NPs significantly restored fruit quality and productivity. The dual treatment with BCF and 200 ppm ZnO-NPs produced the best performance under pathogen pressure, with fruit length of 17.0 ± 0.71 cm, fruit diameter of 3.9 ± 0.07 cm, average fruit weight of 119 ± 1.8 g, number of fruits per plant of 14.2 ± 0.45, and total yield of 2.42 ± 0.08 kg—significantly higher than other infected treatments (*p* ≤ 0.05).

These results demonstrate a clear synergistic effect between BCF and ZnO-NPs in enhancing cucumber fruit yield and quality, with particularly strong mitigation of *Pantoea*-induced losses.

Foliar application of *Bacillus* culture filtrate (BCF) and zinc oxide nanoparticles (ZnO-NPs) markedly influenced the fruit quality attributes of cucumber at harvest (Table 7). In the absence of *Pantoea* sp. infection, increasing ZnO-NP concentrations progressively enhanced total soluble solids (TSS), vitamin C, and total carotenoids content, with the highest values recorded at 200 ppm ZnO-NPs combined with BCF (5.10 ± 0.12°Brix, 7.10 ± 0.19 mg/100 g FW, and 0.63 ± 0.03 mg g^−1^ FW, respectively). Correspondingly, titratable acidity (TA) and pH also increased slightly with the treatments, reaching 0.43 ± 0.03% CA and 6.44 ± 0.10 at 200 ppm ZnO-NPs + BCF.

**Table 7:** Fruit quality parameters of cucumber at harvest as influenced by foliar application of *Bacillus* culture filtrate (BCF) and zinc oxide nanoparticles (ZnO-NPs), with or without *Pantoea sp.* infection

BLS Infection	BCF Treatment	ZnO-NPs (ppm)	Total soluble solids (°Brix)	Vitamin C (mg 100 g^−1^ FW)	pH	Titratable acidity (%CA)	Total carotenoids (mg g^−1^ FW)	Cucurbitacins (mg kg⁻^1^ DW)
Without	- BCF	0	2.83 ± 0.21 h	3.06 ± 0.15i	5.33 ± 0.06 g	0.15 ± 0.01i	0.20 ± 0.01jk	0.12 ± 0.01d
		50	3.18 ± 0.10 g	4.33 ± 0.20f	5.53 ± 0.11f	0.33 ± 0.02e	0.33 ± 0.02 g	0.05 ± 0.01f
		100	4.18 ± 0.12d	5.44 ± 0.30d	5.69 ± 0.09e	0.36 ± 0.01d	0.47 ± 0.02d	0.04 ± 0.01gh
		200	4.96 ± 0.16ab	6.82 ± 0.11b	6.35 ± 0.11a	0.42 ± 0.02ab	0.62 ± 0.02ab	0.02 ± 0.01hi
	+ BCF	0	4.01 ± 0.22e	4.03 ± 0.18 g	5.64 ± 0.09e	0.21 ± 0.01 h	0.24 ± 0.02i	0.09 ± 0.02e
		50	4.07 ± 0.15de	4.83 ± 0.19e	5.82 ± 0.08d	0.30 ± 0.01f	0.40 ± 0.01ef	0.04 ± 0.01 fg
		100	4.63 ± 0.10c	6.19 ± 0.19c	6.10 ± 0.12b	0.40 ± 0.02c	0.54 ± 0.03c	0.03 ± 0.01ghi
		200	5.10 ± 0.12a	7.10 ± 0.19a	6.44 ± 0.10a	0.43 ± 0.03a	0.63 ± 0.03a	0.02 ± 0.01i
With	- BCF	0	1.60 ± 0.07j	2.00 ± 0.13j	5.00 ± 0.07i	0.09 ± 0.01 k	0.10 ± 0.01 l	0.22 ± 0.01a
		50	2.01 ± 0.14i	2.95 ± 0.16i	5.07 ± 0.05hi	0.17 ± 0.02i	0.22 ± 0.02ij	0.19 ± 0.01b
		100	2.79 ± 0.09 h	3.55 ± 0.11 h	5.44 ± 0.11f	0.24 ± 0.02 g	0.37 ± 0.01f	0.13 ± 0.02d
		200	3.49 ± 0.08f	5.27 ± 0.12d	5.98 ± 0.11c	0.37 ± 0.01d	0.54 ± 0.04c	0.09 ± 0.02e
	+ BCF	0	2.03 ± 0.11i	2.13 ± 0.11j	5.11 ± 0.12 h	0.12 ± 0.01j	0.17 ± 0.01 k	0.19 ± 0.01b
		50	2.74 ± 0.10 h	3.05 ± 0.15i	5.52 ± 0.08f	0.20 ± 0.01 h	0.29 ± 0.02 h	0.15 ± 0.02c
		100	3.43 ± 0.12f	4.14 ± 0.11 fg	5.72 ± 0.07e	0.32 ± 0.02e	0.41 ± 0.02e	0.11 ± 0.01d
		200	4.84 ± 0.11b	6.74 ± 0.11b	6.18 ± 0.13b	0.41 ± 0.02bc	0.60 ± 0.04b	0.03 ± 0.01ghi

*BLS* bacterial leaf spot caused by *Pantoea* sp.; BCF: *Bacillus* culture filtrate, where (–BCF) indicates absence and (+ BCF) indicates presence. Values represent as mean ± SD (*n* = 5).Means within each column followed by the same alphabetical letter(s) do not differ significantly at *p* ≤ 0.05(*)

Under *Pantoea* sp. infection, fruit quality parameters were generally reduced compared with the healthy control; however, application of BCF and ZnO-NPs significantly mitigated these reductions. The combined treatment (BCF + ZnO-NPs) notably restored TSS, vitamin C, and carotenoids levels in infected plants, achieving 4.84 ± 0.11°Brix, 6.74 ± 0.11 mg/100 g FW, and 0.60 ± 0.04 mg g^−1^ FW, respectively, at 200 ppm ZnO-NPs.

Cucurbitacin accumulation (mg kg^−1^ DW) followed an inverse pattern, decreasing with increasing ZnO-NP concentration and BCF treatment, particularly under *Pantoea* sp. stress, where the combined treatment reduced cucurbitacin content to 0.03 ± 0.01 mg kg^−1^ DW compared with 0.22 ± 0.01 mg kg^−1^ DW in untreated infected plants.

These results indicate that foliar-applied ZnO-NPs, especially in combination with BCF, enhanced the nutritional and biochemical quality of cucumber fruits while simultaneously reducing the stress-induced accumulation of cucurbitacins under *Pantoea* sp. infection.

## Discussion

Zinc oxide nanoparticles (ZnO-NPs) are inorganic nanofertilizers classified as “generally recognized as safe” and widely applied in crop production and plant disease control [66]. Some *Bacillus* species are well-documented plant growth-promoting rhizobacteria (PGPR) known to enhance plant growth and induce systemic resistance through the production of diverse bioactive metabolites [14]. The use of *Bacillus* culture filtrates, rather than live inocula, represents an eco-friendly and practical alternative that mitigates issues related to microbial persistence, ecological imbalance, and inconsistent colonization efficiency.

In vitro assays confirmed strong antibacterial activity of both ZnO-NPs and *Bacillus* culture filtrate (BCF) against *Pantoea* sp., with ZnO-NPs showing a clear dose–response relationship and maximum inhibition (38 mm) at 200 ppm. This aligns with reports attributing ZnO-NPs’ activity to reactive oxygen species generation, zinc ion release, and bacterial membrane disruption [67, 68]. Their nanoscale size and large surface area facilitate interaction with bacterial cell walls, leading to structural breakdown, genetic damage, protein malfunction, and cell death [69, 70]. The BCF exhibited a 40 mm inhibition zone, consistent with the presence of volatile antimicrobial metabolites identified in Table 2. These compounds are known to disrupt bacterial membrane integrity and interfere with essential enzymatic functions [71, 72]. The complementary antibacterial mechanisms—ZnO-NPs–induced oxidative stress and BCF metabolite–mediated antagonism—suggested a potential synergistic effect, which was subsequently validated under greenhouse conditions. This is consistent with previous reports demonstrating synergistic interactions between nanoparticles and microbial biostimulants in the integrated management of biotic and abiotic stresses [73–75]. Metabolite profiling of BCF further supported these observations, revealing multiple bioactive compounds, including oleic acid, cis-13-octadecenoic acid, palmitic acid, and various long-chain fatty acid methyl esters, all reported for antibacterial, antioxidant, and growth-regulating properties [52–64]. The presence of these compounds provides a molecular basis for the observed in vitro suppression of *Pantoea* sp.

Greenhouse trials confirmed that combining ZnO-NPs and BCF enhanced disease suppression beyond individual applications. The pronounced reduction in disease severity with ZnO-NPs reflects their antimicrobial action [76], while the enhanced suppression in the combination likely originated from *Bacillus* sp. lipopeptides, antibiotics, and defense-eliciting molecules [77]. This is consistent with earlier studies showing enhanced pathogen control through nanomaterial–microbe integration [73–75]. The minimal disease severity recorded with 200 ppm ZnO-NPs + BCF (1.1 ± 0.14) underscores the strength of this interaction, comparable to disease control observed in beetroot with ZnO-NPs [78].

Beyond disease suppression, both ZnO-NPs and BCF improved cucumber vegetative growth under healthy and infected conditions. ZnO-NPs alone have been shown to enhance nutrient uptake, photosynthesis, and antioxidant defense [79–81], while BCF metabolites can prime plant defense and growth responses. Under pathogen pressure, untreated plants exhibited significant growth reduction, but combined treatments maintained growth parameters at or above healthy controls. These results reflect previous findings on *Bacillus*-induced systemic resistance [82, 83] and ZnO-NP–mediated stress mitigation [81].

Nutrient analysis further clarified this synergy. The 200 ppm ZnO-NPs + BCF treatment consistently yielded the highest N, P, K, Zn, and Fe levels in both healthy and infected plants. ZnO-NPs likely enhanced membrane permeability and root activity [84], and promoted nitrogen metabolism via nitrate reductase activation [85, 86]. Meanwhile, BCF may have facilitated mineral mobilization through organic acid and siderophore production [87]. Under infection, nutrient depletion in controls contrasted sharply with the restored profiles in treated plants, reflecting the combined nutritional, antimicrobial, and stress-alleviating roles of ZnO-NPs and BCF [67–72].

Biochemical and physiological parameters reinforced this pattern. Both agents increased total phenolic content and chlorophyll (SPAD values), with the combination at 200 ppm ZnO-NPs producing the highest levels under both healthy and infected conditions. Elevated phenolics indicate activation of plant defense pathways, particularly under pathogen stress [88, 89], while higher SPAD values suggest improved photosynthetic performance due to zinc’s role in chlorophyll synthesis [80] and BCF’s capacity to enhance nutrient status and reduce oxidative damage [90]. The complementary actions—BCF inducing systemic resistance and ZnO-NPs supplying nutritional and antimicrobial support—likely underlie the observed synergy [88, 89, 91].

The observed improvements in fruit yield and quality following ZnO-NP and BCF application can be attributed to the synergistic interaction between the biostimulatory properties of ZnO-NPs and the plant growth-promoting metabolites of BCF. Zinc plays pivotal enzymatic, hormonal, and structural roles in plants [92–95], while BCF provides bioactive secondary metabolites that stimulate physiological processes [95]. Even under pathogen infection, yield parameters approached those of healthy controls, underscoring the protective advantage of this integrated approach.

The present study further demonstrated that both BCF and ZnO-NPs, whether applied individually or in combination, significantly enhanced cucumber fruit quality under both healthy and BLS-infected conditions. The combined application of BCF with 200 ppm ZnO-NPs resulted in the highest total soluble solids (°Brix), ascorbic acid, and carotenoid contents, coupled with the lowest cucurbitacin accumulation—thereby improving the nutritional, sensory, and commercial quality of cucumber fruits. The marked increases in °Brix and vitamin C indicate enhanced metabolic activity and photoassimilate translocation, consistent with previous findings on nanofertilizer- and *Bacillus*-based treatments [45].

The elevated carotenoid content also reflects improved antioxidant potential, contributing to superior nutritional value and extended postharvest longevity, in line with previous reports [96]. Moreover, the significant reduction in cucurbitacins observed under the combined treatment highlights improved fruit palatability and consumer acceptance—key indicators of cucumber quality [97].

Overall, the concurrent enhancement of fruit quality, growth, and yield suggests an integrative physiological response involving improved nutrient assimilation, hormonal balance, and stress alleviation, as supported by recent studies on nanotechnology and microbial biostimulants in sustainable vegetable production [98, 99].

Collectively, these results indicate that integrating *Bacillus* culture filtrates with ZnO-NPs offers a sustainable, eco-friendly strategy for controlling *Pantoea* leaf spot while enhancing cucumber growth, nutrition, physiology, and yield. The proven synergy between nanotechnology and beneficial microbes warrants field-scale validation under diverse environmental conditions to confirm its practical applicability.

## Conclusions and future perspectives

This study highlights the synergistic potential of *Bacillus* culture filtrate and zinc oxide nanoparticles (ZnO-NPs) in suppressing *Pantoea* sp., the causal agent of bacterial leaf spot in cucumber, under greenhouse conditions. The combined treatment significantly reduced disease severity, improved physiological performance, enhanced nutrient uptake, and increased fruit yield compared with individual applications. These findings indicate that integrating *Bacillus*-derived metabolites with ZnO-NPs can contribute to disease suppression and growth promotion through complementary antimicrobial and physiological effects.

GC–MS profiling of the *Bacillus* sp. culture filtrate revealed diverse bioactive metabolites that may play important roles in pathogen inhibition and plant defense activation. Further studies are required to confirm these mechanisms and to determine their relevance under field conditions.

Overall, the co-application of ZnO-NPs and *Bacillus* culture filtrate represents a promising, environmentally sustainable approach to improving crop health. Future research should focus on mechanistic elucidation, formulation optimization, and large-scale validation to support its potential application in sustainable crop protection.

## Data Availability

The DNA sequence data generated in this study have been deposited in the NCBI GenBank database under accession numbers PX509262 (*Pantoea* sp.) and PX509261 (*Bacillus* sp.). These sequences are publicly available at https://www.ncbi.nlm.nih.gov/genbank/.
